# Placebo Analgesia Does Not Generalize From Pain to Interoceptive Abilities: A Preregistered Exploratory Study

**DOI:** 10.1111/psyp.70391

**Published:** 2026-08-29

**Authors:** Helena Hartmann, Alessio Proposito, Federica Riva, Claus Lamm

**Affiliations:** ^1^ Social, Cognitive and Affective Neuroscience Unit, Department of Cognition, Emotion, and Methods in Psychology, Faculty of Psychology University of Vienna Vienna Austria; ^2^ Department for Neurology and Center for Translational Neuro‐ and Behavioral Sciences (C‐TNBS) University Hospital Essen Essen Germany; ^3^ Donders Institute for Brain, Cognition and Behaviour Radboud University Medical Center Nijmegen the Netherlands; ^4^ Vienna Cognitive Science Hub University of Vienna Vienna Austria

**Keywords:** heartbeat perception, heartbeat tracking, interoception, pain, placebo analgesia

## Abstract

Placebo pills reliably reduce pain. Pain perception is tied to increased arousal and heart rate. Heart rate perception is, in turn, closely related to interoceptive signals processing, for example, pain and autonomic regulation. However, no studies so far have systematically examined the generalizing versus specific effects of placebo analgesia in transferring from pain to the interoception of cardiac signals. In this preregistered, exploratory study, we aim to investigate whether the placebo analgesia effect on pain may generalize to the perception of one's own heartbeat. We recruited 88 healthy participants (47 female, 41 male), of which half (23 female, 21 male) underwent a deceptive placebo analgesia induction. Using the Heartbeat Counting Task and the MAIA questionnaire, we derived three interoceptive dimensions: interoceptive accuracy, sensibility (at both task‐related and trait levels), and awareness. Despite a robust placebo analgesia effect, no difference between the placebo and the control group was found in any of the interoceptive dimensions. These findings were supported by post hoc Bayesian analyses, which indicated moderate evidence for this absence of a group difference. Our findings thus do not indicate a generalization of the placebo analgesia effect from pain to cardiac interoceptive awareness. Studies such as the present one are important to better understand the perception of different bodily systems and their connection in humans.

## Impact Statement

Can a top‐down reduction of pain perception also influence the perception of other bodily signals, such as the heartbeat? This study systematically examined the generalizing versus specific effects of placebo analgesia in transferring from pain perception to the interoception of cardiac signals. We show moderate evidence of absence for such transfer effects using the heartbeat counting task.

## 1 Introduction

Pain is defined as an unpleasant sensory and emotional experience associated with actual or potential tissue damage (Loeser and Treede 2008). Pain sensations are directed to the central nervous system through the lamina I spinothalamocortical system. This circuit is also responsible for delivering other physiological sensations to the brain, such as temperature, itch, muscular, and visceral sensations (Craig 2002). This system therefore seems to be a common peripheral pathway underlying all interoceptive sensations (Li et al. 2017), which are crucial to regulate bodily homeostasis (Khalsa et al. 2018).

Interoception is distinguished from exteroception, which refers to the perception of the external environment, and proprioception, the sensing of the position of the body in space (Craig 2007; Garfinkel et al. 2015). Interoception is a complex construct that can be operationalized using different dimensions. Regarding conscious interoception, Garfinkel et al. (2015) proposed a model entailing three different components: interoceptive accuracy, interoceptive sensibility and interoceptive awareness. The first one refers to the *objective* ability to correctly perceive our own bodily states, while the second one indicates the *subjective* belief about our own interoceptive accuracy. The third one represents a meta‐cognitive measure of the correspondence between the first two measures, although studies show that these accuracy and awareness dimensions only show weak to moderate associations and might be distinct from each other (e.g., Köteles et al. 2020).

Indeed, painful sensations are often associated with increases in other physiological responses relevant for interoception, such as skin conductance, arousal and heart rate (Kyle and McNeil 2014; Loggia et al. 2011; Treister et al. 2012; del Paso et al. 2011; Wegeberg et al. 2024 for a review). In the field of pain, “bottom‐up” processes refer to nociceptive input originating in peripheral tissues (e.g., skin or muscles) and ascending through the spinal cord to the brain (Tiemann et al. 2015). These signals reflect physical stimulus properties, such as intensity or tissue damage, crucially shape the complex subjective pain experience. In contrast, “top‐down” processes involve cognitive and affective influences (e.g., expectations or prior experiences). Those originate in the brain and modulate pain processing at cortical and subcortical levels, including descending modulation of spinal nociceptive transmission (Bingel 2020). Placebo analgesia, defined as pain reduction through inert, nonpharmacological treatments, has been extensively documented (Colloca et al. 2013; Colloca and Benedetti 2006). Next to reliably reducing pain perception, placebo analgesia has also been shown to have downstream emotional effects on empathy for other people's pain (Rütgen, Seidel, Riečanský, and Lamm 2015; Rütgen, Seidel, Silani, et al. 2015), first‐hand perception of and empathy for unpleasant touch (Rütgen et al. 2021), and even influence physical effort exerted to support others in pain (Hartmann et al. 2022). Importantly though, all of these findings relate to negative sensations, while no generalization was observed regarding pleasant touch sensations (Rütgen et al. 2021). These studies also mostly focused on higher‐order processes shaping the subjective and neuronal experience of pain, but it is unclear whether such cognitive modulation of pain may generalize to other (autonomic nervous system) bodily signals, especially those entwined with the perception of pain, like heartbeat. This is highly relevant in light of the still ongoing opioid crisis in many countries (Gardner et al. 2022; Volkow and Blanco 2021). What other transfer effects do decreased pain sensations and top‐down modulation of pain have on our perception? What effects does this have for the correct and fast evaluation of our homeostatic state and health level? Can expectations about one bodily sensation influence another one?

While causal studies are still absent, the correlative association between pain and interoception has been investigated in a few studies (Ferentzi et al. 2021; Pollatos et al. 2012; Weiss et al. 2014). Prior work suggests that enhanced sensitivity and decreased tolerance to pain in healthy subjects are associated with higher state interoceptive accuracy (Pollatos et al. 2012). Reduced interoceptive scores on a cardiac task were found in somatoform patients who showed stronger pain tolerance, compared to controls (Weiss et al. 2014). These findings point towards a connection between one's ability to perceive pain signals and one's interoceptive abilities. On the other hand, studies such as Werner et al. (2009) or Ferentzi et al. (2021) report pain and interoception to be at least partially independent. Rütgen et al. (2021) provided causal evidence that placebo analgesia generalizes to reducing emotions associated with unpleasant touch (see also Rütgen and Lamm 2024, for a recent review). The authors also found that the manipulation reduced neural activity in the bilateral insular cortex. The insular cortex is, in turn, widely considered a crucial hub for the processing of interoceptive signals and their awareness (Caseras et al. 2013; Critchley et al. 2004; Ernst et al. 2013; Pollatos et al. 2007; Stern et al. 2017; Tan et al. 2018; Wiebking et al. 2014, 2015; Zaki et al. 2012). However, it is still unclear whether a top‐down decrease of pain perception, as induced with placebo analgesia, influences a bottom‐up interoceptive ability such as cardiac perception.

In this preregistered study, we hypothesized that the pain‐reducing effects of placebo analgesia generalize to heartbeat perception. Given the limited, prior evidence on the causal relationship between placebo analgesia and interoceptive abilities, we did not make directional predictions in our preregistration as to the nature of this generalization. Instead, we formulated two competing (and thus exploratory) outcomes in our preregistration: (1) Participants under placebo analgesia might show reduced interoceptive abilities, for example due to a transfer of the analgesic effect to a general reduction in the perception of bodily sensations; (2) Participants under placebo analgesia might show increased interoceptive abilities, for example due to the knowledge of having taken pain medication and thus enhanced focus on one's interoceptive signals (e.g., via side effect alertness; Webster et al. 2016).

## 2 Materials and Methods

### 2.1 Preregistration

This study was preregistered on the OSF prior to any creation of data and included the study design and an analysis plan (https://doi.org/10.17605/OSF.IO/GH4SF). In the methods and results, we clearly separate preregistered procedures and analyses from those added post hoc. A pilot study with nine subjects was conducted before the upload of the preregistration and the start of data collection to test the study procedure, especially for the placebo group. However, this data was not used in the final analysis. The data and code needed to run all analyses and reproduce the figures can be found on our Open Science Framework (OSF) project (Hartmann et al. 2019; https://osf.io/fz73k/). We report how we determined our sample size, all data exclusions, all manipulations, and all measures in the study.

### 2.2 Participants

This project was part of a bigger study conducted with multiple tasks, where the interoception task was performed last, which is why the procedures described in the following are equal to those in (Hartmann et al. 2022). The latter study reported on the effects of placebo analgesia on empathy for pain and prosocial behavior, while this manuscript focuses on the effects of the same placebo analgesia manipulation on interoception.

Each participant was first screened for exclusion criteria using a thorough online questionnaire sent via email. Exclusion criteria were a past or present enrolment in academic studies including psychology, pharmaceutics or medicine (veterinary, dental, human, etc.) as well as other medical‐related training (e.g., nurse), any neurological or psychiatric conditions, chronic conditions or conditions related to the central nervous system (CNS), past or present substance abuse (alcohol more than daily use, drugs weekly or daily use), the intake of psychopharmacological medication (besides oral contraceptives) within the last 3 months, and past participation in at least one placebo study or a study involving similar deceptive elements. Furthermore, participants above the clinical cut‐offs of the following questionnaires were excluded to create a more homogenous, neurotypical sample: Beck's Depression Inventory II (BDI‐II; cut‐off = 14 (Kühner et al. 2007), *n*_excluded_ = 5); Autism Quotient (AQ‐k; cut‐off = 17 (Freitag et al. 2015), *n*_excluded_ = 1); Toronto Alexithymia Scale (TAS‐20; cut‐off = 51 (Bach et al. 1996), *n*_excluded_ = 5).

Our preregistered target sample size was 90 participants (45 participants per group), excluding placebo analgesia non‐responders and participants expressing doubts about the deceptive elements of the study. Precise a priori power calculations were not possible due to the absence of previous studies investigating our research question and thus a lack of fitting effect size estimates. We therefore based our group sizes on previous studies using placebo analgesia in a between‐subjects design to investigate transfer effects on empathy (Rütgen, Seidel, Riečanský, and Lamm 2015; Rütgen, Seidel, Silani, et al. 2015). Once deemed eligible, participants were pseudorandomly allocated to either the placebo or control group. A document with subject codes and group allocation was created beforehand, where placebo and control sessions were alternated (Subject_01 = control, Subject_02 = placebo, Subject_03 = control, etc.). Dropouts in each group were replaced and added at the end of this document until the final group sizes were reached. We recruited a total of 124 participants, 34 of whom were excluded during data collection due to procedures described in Hartmann et al. (2022) relating to another task (e.g., because of doubts about the cover story or thoughts about having received a placebo). Two additional participants were not included in the analyses described here because of missing electrocardiogram (ECG) data during the performance of the interoceptive task. The two groups were matched closely regarding age (mean age ± standard deviation = 23.54 ± 2.94 (placebo group) and 24.02 ± 4.37 (control group)) (*t*(86) = −0.60, *p* = 0.549), gender (*χ* ^2^(1) = 0.00, *p* = 1.000), body mass index (𝑡(86) = −0.52, *p* = 0.603) and perceived trait interoceptive abilities (mean total score ± standard deviation = 4.07 ± 0.54 (placebo group) and 4.04 ± 0.58 (control group)) (*t*(86) = 0.25, *p* = 0.799), measured with the German version of the Multidimensional Assessment of Interoceptive Awareness (MAIA) (Mehling et al. 2012). Participants were neurotypical, German‐speaking, right‐handed young adults with normal or corrected‐to‐normal vision who were currently enrolled in an Austrian university (Table 1). The final sample included *n* = 88 participants (placebo group: *n* = 44; control group: *n* = 44). The present study included no patient or public involvement.

**TABLE 1:** Overview of the sample and its characteristics.

	Overall (*N* = 88)	Placebo (*N* = 44)	Control (*N* = 44)	Group comparison
*Gender (F = female, M = male)*				
Frequency	F = 47, M = 41	F = 23, M = 21	F = 24, M = 20	*χ* ^2^(1) = 0.00, *p* = 1.000
*Age (years)*				
Mean (SD)	23.78 (3.71)	23.54 (2.94)	24.02 (4.37)	𝑡(86) = −0.60, *p* = 0.549
*Body Mass Index*				
Mean (SD)	22.8 (3.04)	22.62 (2.55)	22.97 (3.48)	𝑡(86) = −0.52, *p* = 0.603
*Education level*				
Frequency	1 = 1 2 = 57 3 = 22 4 = 8	1 = 1 2 = 25 3 = 15 4 = 3	1 = 0 2 = 32 3 = 7 4 = 5	*χ* ^2^(3) = 5.27, *p* = 0.153

*Note:* The placebo and the control groups included the same number of participants and showed no difference in age, gender, education, and BMI. Two‐sample *t*‐tests were used to test group differences in age and BMI, and a chi‐squared test to test if the gender and education level distribution differed in the placebo and control groups. The different levels of education were categorized in this way: (1) vocational training, (2) high school diploma, (3) Bachelor's degree, and (4) Master's degree or higher. F, female; M, male.

### 2.3 Procedure

We conducted a cross‐sectional, experimental study. Eligible participants answered questionnaires online prior to coming to the testing session in the laboratory. They were asked to refrain from alcohol, drugs and medication intake (except oral contraceptives) in the 24 h leading up to the experiment and to refrain from smoking, food, caffeine, and any drinks other than water 1 h before the experiment. The behavioral testing session started with general instructions and calibration of individual pain threshold (see below for thorough descriptions of each step). Depending on the group, this was either followed by the placebo induction (placebo group) or an equal waiting time (control group). After completing another task unrelated to this study, participants completed the heartbeat counting task on a computer alone in a quiet chamber (i.e., around 45 min after the placebo induction). Small breaks of a few minutes were kept between different steps and tasks. The session ended with follow‐up questions and feedback from the participants, which also served as a recovery period of around 15 min. The whole experiment lasted around 3 h and participants received 35€ for their participation. The local ethics committee of the University of Vienna approved all procedures beforehand (application number 00412). The present research was conducted in accordance with the 2013 Declaration of the World Medical Association and all participants gave written consent before participation and before any data was collected. Participants were explicitly told that they could discontinue their participation at any time without negative consequences and were debriefed about all deceptive elements upon conclusion of the study.

#### 2.3.1 Pain Calibration

The pain calibration was identical to the one described in (Hartmann et al. 2022) where 500 ms electrical stimulations were given to the dorsum of the non‐dominant, left hand in multiple trials, moving up stepwise from a rating from 0 = “not perceivable” until the stimulus was rated as 8 = “extremely painful but bearable” (with a rating of 7 indicating very painful, but still bearable pain). The experimenter started by delivering stimuli at a low intensity of 0.05 mA, with the step size individually chosen for each participant, so that each rating was experienced at least once. This was repeated a second time with a small break in between to avoid habituation/sensitization and followed by varying (seemingly “random”) stimulation in the before calibrated rating range from 1 to 8. This procedure allowed us to gain valid averages for values the participant rated as 1 = “noticeable but not painful,” 4 = “medium painful,” and 7 = “very painful.” Average intensities rated as 1 and 7 were used in the pain task for non‐painful and painful stimulation, respectively. Participants were told that they would receive stimulation in the following tasks that they had rated as very painful before, without providing exact numbers.

#### 2.3.2 Placebo Analgesia Induction

The placebo group underwent a deceptive placebo analgesia induction well validated from previous studies (e.g., Rütgen, Seidel, Riečanský, and Lamm 2015; Rütgen, Seidel, Silani, et al. 2015). This procedure combined verbal suggestions by a medical student (presented as a doctor working at the Faculty of Psychology) and, after a waiting time of 10 min for the pill “to take effect”, a conditioning procedure in regard to the treatment. The verbal suggestions involved telling the participant that they would ingest an “effective and powerful painkiller” in pill form which has already been shown to reliably reduce pain in multiple studies. We were, however, interested in its effects on different computer tasks. We opted for an external study doctor in a white lab coat instead of the main experimenter to administer the placebo treatment to increase believability and boost the placebo effect. The conditioning procedure was employed to further increase the placebo effect through direct, suggested experience of pain relief. It involved the participants receiving electrical stimulation in the same location as during calibration. Participants were told they would receive high stimulation intensities they rated as “very painful” before, but in reality covertly received stimulation of a medium intensity (rated as 4 before) and, after giving their rating, corresponding feedback (e.g., “This stimulus was rated as more painful/very painful before”) to suggest a pain reduction by means of the given treatment. These trials were repeated for a minimum of two and up to a maximum of four times until the subject did not respond with values higher than 5 to the conditioning stimulus, that is, they experienced a pain reduction. In reality, the pill did not contain any active pharmacological components, but a natural sugar‐alcohol (Mannitolum, 40G per pill). The control group did not receive a pill or any type of induction but had equally long waiting times to keep the session length the same.

Two inherent characteristics of deceptive placebo studies are (1) the absence of blinding regarding the placebo/control conditions and (2) the building of positive expectations regarding the alleged treatment (e.g., pain relief). Blinding of participants or experimenters regarding the placebo/control conditions was therefore not intended, as both parties needed to know this information for the placebo analgesia manipulation to exert maximal effects. This approach is common and well‐established practice in studies that focus on the mechanisms and processes of placebo analgesia (e.g., Blythe et al. 2023; Forsberg et al. 2017; Meissner and Linde 2018; Zunhammer et al. 2021), and differs from double‐blind paradigms in clinical trials which have the different aim of controlling for rather than inducing placebo analgesia (Vase et al. 2002). To minimize differences between experimenters and ensure that all participants received exactly the same information and cover story, all instructions were read verbatim from a pre‐specified script.

#### 2.3.3 Pain Task

The pain task was a shortened version of the one used in (Rütgen, Seidel, Silani, et al. 2015) with a total of 20 trials (5 per condition) and the trials where the participants received pain first‐hand were used as a manipulation check to ensure that we had reduced pain in the placebo group through the treatment. The original task also contained an empathy condition that is reported in (Hartmann et al. 2022). This task was completed right before the Heartbeat Counting Task. Trials were shown in a pseudorandomized order (four sequences were created manually for each task and alternated over all participants in an a priori defined order). After every trial, we asked participants to evaluate: “How painful was this stimulation for you?” on a 9‐point rating scale from 0 = “not noticeable” to 8 = “extremely painful/unpleasant.”

#### 2.3.4 Heartbeat Counting Task

To measure interoceptive abilities, we used the classic Heartbeat Counting Task (HCT; Schandry 1981; but see Ferentzi et al. 2022). In this task, participants are asked to perceive their own heartbeat and count the number of beats in specified time intervals. The counted number is then compared to the actual number of measured heartbeats, which yields an interoceptive accuracy score. The accuracy is also compared to a control condition of exteroceptive perception, such as counting tones. We therefore employed an interoceptive condition (counting one's own heartbeat) and an exteroceptive control condition (counting tones heard over headphones). Importantly, and in response to the criticism about the HCT, we used modified instructions that underlined to the participants that they should not use any help while counting their heartbeat (like feeling the pulse), and that they should only report the beats they really felt and under no circumstances guess or estimate (Corneille et al. 2020; Desmedt et al. 2018). This could mean reporting no beat at all, some beats, or as many beats as were actually counted.

We continuously measured the actual heart rate via ECG to identify the number of heartbeat peaks in each trial of the interoceptive condition using Mobi, a measurement system for electrophysiological signals (Twente Medical Systems International, Netherlands). To this end, participants were equipped with electrodes to measure electrocardiac signals during the task. Three electrodes were placed on non‐hairy/shaved skin of the insides of each ankle and the left inside wrist, which had been cleaned with water and rubbed with a towel to increase conductivity. The electrode on the right leg functioned as the ground electrode. Participants were then asked to breathe strongly for a few seconds to check the strength of the ECG signal. The experimenter continued with the procedure if a discernible heart rate was visible on the screen; if not, the electrodes were reattached until this was the case.

**FIGURE 1:**
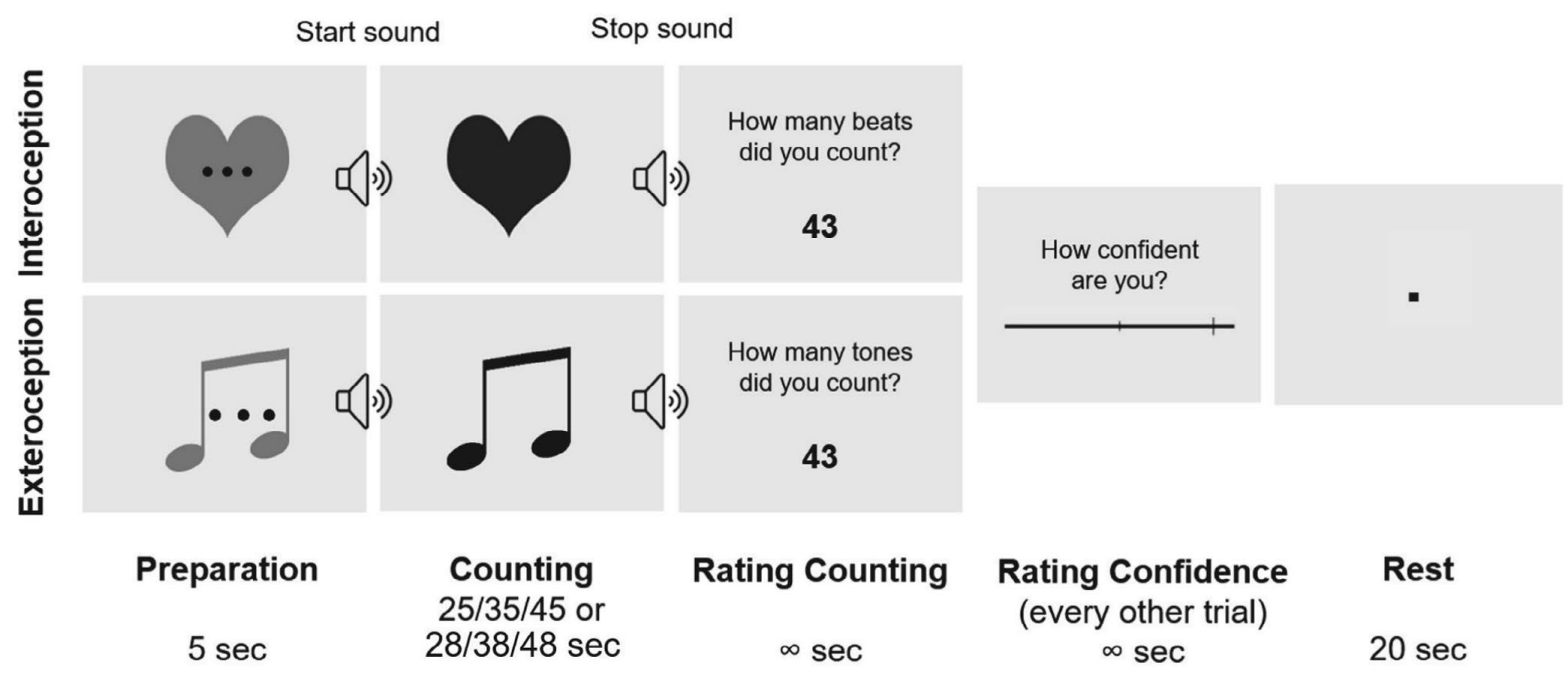
Overview of the heartbeat counting task. Participants either counted their own heartbeats or tones audible over headphones and then reported the number of beats/tones that they counted and, every other trial, also their confidence in their reported number.

Participants then completed a training, starting with a 5‐min resting phase. During this phase, participants were asked to sit comfortably in their chair, close their eyes and concentrate on their heartbeat while relaxing. This was followed by a sound calibration to adjust the volume of the tones heard later in the task to make the exteroceptive and interoceptive conditions as equally difficult as possible. A set of tones of increasing or decreasing volume was played while participants wore headphones. Participants were asked to immediately press a button when they (1) heard the tones for the first time (volume increase) or (2) did not hear the tones anymore (volume decrease) (order: decrease—increase—decrease—increase). An average volume was calculated from these four trials and after that, 10 single tones were played in this average volume, with the participant indicating for each of them whether they had heard a tone or not. The participant was told that sometimes a tone would be played and sometimes not. If the participant perceived 9 or 10 tones, the average volume was decreased by two steps (chosen volume step = 0.5% of the maximum volume); if the participant perceived 7 or 8 tones, the average volume was decreased by one step; if the participant perceived 4, 5, or 6 tones (i.e., 40%–60% of all tones), the average volume was not changed; if the participant perceived 2 or 3 tones, the average volume was increased one step; and if the participant perceived 0 or 1 tone(s), the average volume was increased by two steps. Then again, five single tones were played in the same fashion in the adjusted volume and another correction was applied, depending on the number of total tones perceived: If the participant perceived 5 tones, the average volume was decreased by two steps; if the participant perceived 4 tones, the average volume was decreased one step; if the participant perceived 2 or 3 tones (= 40%–60% of all tones), the average volume was not changed; if the participant perceived 1 tone, the average volume was increased one step; and if the participant perceived 0 tones, the average volume was increased two steps. The resulting volume was used in the following training and task (see Figure 1).

Then, participants completed a short practice (4 trials, each condition twice) and then the main task with 12 trials, 6 for each condition (interoceptive, exteroceptive), using 3 different trial durations. The durations differed for the two conditions to avoid transfer/learning (e.g., participants remembering the amount of beats from a previous trial) from one condition to the other (durations were 25/35/45 s and 28/38/48, respectively, and were counterbalanced between subjects). Icons on the screen indicated the upcoming condition in the next trial to the participant. After each trial, participants were asked to report the number of heartbeats or tones they counted, followed by a brief rest period of 20 s before the next trial. In every other trial, participants were also asked how confident they felt about their given answer (to measure interoceptive sensibility) on a continuous visual analogue scale (200 points) with the anchors “not confident” and “confident”. Our approach therefore assessed (1) performance in accuracy and (2) subjective beliefs about it (Murphy et al. 2019). Trials of the interoception task were shown in a pseudorandomized order (four sequences were created manually for each task and alternated over all participants in an a priori defined order).

We then calculated interoceptive accuracy scores by comparing the subject's reported number of heartbeats with the actual number of heartbeats recorded via ECG during the same intervals. Accuracy was computed for each participant using the standard formula: 1/total number of time intervals Σ (1–(|actual heartbeats—reported heartbeats|)/actual heartbeats). In this formula, the normalized absolute difference between reported and actual heartbeats was subtracted from 1, yielding scores ranging between 0 and 1, with higher numbers representing higher accuracy. The same formula was used for the exteroceptive condition. Interoceptive sensibility scores were calculated as a mean of all confidence ratings of one condition.

### 2.4 Data Acquisition and Analysis

Data were processed and analyzed using RStudio (Version 1.4.17; R Core Team 2020). All *p*‐values were interpreted as two‐sided, as we had two competing hypotheses.

#### 2.4.1 Nonresponder Identification

Non‐responders were determined by procedures equal to the ones used in (Hartmann et al. 2022) and preregistered prior to data collection. In brief, a combination of three measures was employed: First, we recorded any verbally expressed doubts about the treatment or study setup (e.g., confederate or other deceptive parts) by means of feedback questions at the end of the study. If a participant expressed doubts in any of these domains, we asked them to elaborate these further and participants with strong doubts were excluded. Second, differences in the belief scores about the effectiveness of the placebo before and after the induction procedure as well as at the end of the session were analyzed. Here, total belief scores (sum of pre‐ and post‐conditioning scores) lower than 66.6 (values can range from 0 to 200) and strong decreases between the first and second measure (pre‐ score minus post‐conditioning score bigger than 33.3) served as a measure for lack of responding. Third, we took the number of placebo conditioning trials into account. If participants responded with a value greater than 5 on a 9‐point rating scale from 0 = “not noticeable” to 8 = “extremely painful, but bearable” to the conditioning stimulus (delivered at a medium intensity of 4), we deemed the conditioning trial as non‐successful, waited another few minutes for the “medication to take effect”, and then repeated the trial. This was repeated a maximum of four times or until participants responded with values below 6 to the conditioning stimulus in the current trial. If more than three of these trials were necessary, this was taken as an indication of non‐responding. These measures were chosen because they had proven successful in previous studies from our lab and to avoid excluding participants based on their pain ratings within the task (and thus demand characteristics, but see the limitations in the discussion). That also meant that the placebo effects of included participants could still range from none to large, a variability we purposefully wanted to achieve.

#### 2.4.2 Manipulation Checks

2.4.2.1

We operationalized the placebo effect by subtracting the pain rating from the value 7 (i.e., the rating corresponding to the pain intensity delivered, as determined during prior individual pain calibration, see also the mediation analysis in Hartmann et al. 2022), leading to a placebo analgesia score where higher values mean higher analgesia. Some participants who were included based on the preregistered nonresponder criteria could therefore still have scores of 0 or lower, indicating no placebo analgesia effect in the pain rating task. The validity of the placebo analgesia induction on the subjective pain score was assessed with a two‐sample *t*‐test comparing the analgesia scores of the placebo and control group. Exclusively for the placebo group, we asked the participants about their beliefs in the effectiveness of the placebo pill at three different time points of the experiment: after the administration of the pill (pre‐conditioning), after the conditioning procedure and at the end of the session. This was done as a manipulation check on the strength of the placebo effect (not preregistered).

#### 2.4.3 Control Variables

We investigated the comparability of our two groups and included several control measures. These analyses were not preregistered.

##### 2.4.3.1 BMI

Previous studies found a significant effect of the Body‐Mass‐Index on interoceptive abilities, with higher BMI being associated with lower cardiac awareness (Kleckner et al. 2015; Rouse et al. 1988). For this reason, we measured the BMI of participants (using self‐report of weight and height) and included it as a covariate in the two preregistered mixed analysis of variances described in the next section.

##### 2.4.3.2 Clinical Questionnaires

To assess that the placebo and control groups did not differ in any of the clinical questionnaires administered (see Table 2 for the complete list), we performed two‐sample *t*‐tests separately per each of the 9 questionnaires (resulting in 20 tests when including all subscales), and implemented Bonferroni correction for multiple comparisons. In addition, any possible relationship between the clinical questionnaires and the main interoceptive dimensions was investigated with Pearson's correlations, both within the total sample and separately for the placebo and control groups.

##### 2.4.3.3 Post‐Session Questionnaires

During the debriefing at the end of the session, we asked participants how hard versus easy they perceived the interoceptive and exteroceptive condition, respectively, whether they felt the heartbeats in a specific location and/or generally in the body, and to what extent they estimated or guessed when they didn't feel their heartbeat (Desmedt et al. 2018). For each question, participants indicated their answer on a rating scale going from 1 (hard) to 5 (easy) for the first question, and 1 (never) to 9 (always) for the second and third questions. To assess that the two groups did not differ in the evaluation of the difficulty of the two conditions of the HCT, as well as in their strategy to count heartbeats, two‐sample *t*‐tests were used to compare the groups' scores for each of the follow‐up questions.

**TABLE 2:** Overview of the administered behavioral and clinical questionnaires.

Questionnaire	Placebo mean (SD)	Control mean (SD)	Group comparison
IRI			
IRI‐EC	18.36 (4.81)	18.32 (4.79)	𝑡(86) = 0.04, *p* = 0.96
IRI‐PT	18.61 (4.71)	18.27 (3.99)	𝑡(86) = 0.36, *p* = 0.71
HAS	78.64 (8.78)	78.61 (9.61)	𝑡(86) = 0.01, *p* = 0.99
AMI			
AMI‐BA	2.59 (0.6)	2.51 (0.68)	𝑡(86) = 0.61, *p* = 0.54
AMI‐SM	2.87 (0.62)	2.65 (0.71)	𝑡(86) = 1.54, *p* = 0.12
AMI‐ES	6.73 (3.72)	6.70 (2.94)	𝑡(86) = −0.47, *p* = 0.64
SD3‐P	18.34 (3.67)	19.57 (5.39)	𝑡(86) = −1.25, *p* = 0.21
MAIA total	4.07 (0.54)	4.04 (0.58)	𝑡(86) = 0.25, *p* = 0.79
MAIA‐N	4.32 (0.79)	4.56 (0.77)	𝑡(86) = 1.39, *p* = 0.17
MAIA‐ND	3.26 (1.07)	2.95 (0.87)	𝑡(86) = −1.52, *p* = 0.13
MAIA‐NW	3.54 (1.12)	3.64 (1.06)	𝑡(86) = 0.42, *p* = 0.67
MAIA‐AR	3.88 (0.83)	3.92 (0.81)	𝑡(86) = 0.24, *p* = 0.81
MAIA‐EA	4.76 (0.91)	4.62 (0.87)	𝑡(86) = −0.91, *p* = 0.36
MAIA‐SR	3.77 (1.21)	3.70 (1.01)	𝑡(86) = −0.26, *p* = 0.79
MAIA‐BL	3.77 (1.14)	3.49 (1.12)	𝑡(86) = −1.17, *p* = 0.25
MAIA‐T	5.05 (0.95)	5.14 (0.86)	𝑡(86) = 0.41, *p* = 0.68
TAS	38.39 (6.54)	40.11 (6.84)	𝑡(86) = −1.20, *p* = 0.23
AQ	6.73 (3.72)	6.70 (2.94)	𝑡(86) = 0.03, *p* = 0.97
BDI	4.32 (3.7)	4.61 (4.1)	𝑡(86) = −0.36, *p* = 0.72
STAI‐T	36.82 (8.75)	39.48 (7.91)	𝑡(86) = −1.49, *p* = 0.14

*Note:* Placebo and control group did not differ in any of these self‐report measures (all *p*'s > 0.126) before and after applying Bonferroni correction for multiple comparisons.

Abbreviations: AMI, Apathy Motivation Index; AMI‐BA, Behavioral Activation; AMI‐ES, Emotional Sensitivity; AMI‐SM, Social Motivation; AQ, Autism Quotient; BDI, Beck's Depression Inventory; HAS, Prosocial behaviour; IRI‐EC, Interpersonal Reactivity Index—Empathic concern; IRI‐PT, Perspective taking; MAIA, Multidimensional Assessment of Interoceptive Awareness—Trait Sensibility; MAIA‐AR, Attention Regulation; MAIA‐BL, Body Listening; MAIA‐EA, Emotional Awareness; MAIA‐N, Noticing; MAIA‐ND, Not‐Distracting; MAIA‐NW, Not‐Worrying; MAIA‐SR, Self‐Regulation; MAIA‐T, Trust; SD3‐P, Short Dark Triad—Psychopathy; STAI‐T, Trait Anxiety Inventory; TAS, Toronto Alexithymia Scale.

#### 2.4.4 Preregistered Analyses

##### 2.4.4.1 Interoceptive Accuracy (task)

The (interoceptive and exteroceptive) accuracy (IAcc) scores were analyzed with a parametric mixed‐model analysis of variance (ANOVA). As independent variables we included *treatment* (placebo, control) as a between‐subjects factor and *condition* (interoceptive, exteroceptive) as a within‐subject factor. We controlled for *BMI* by including it as a covariate. To check for the presence of ceiling effects (i.e., people having 100% accuracy scores), we adopted a threshold for a minimum of 15% of participants having the highest accuracy value (i.e., 100% or 1) (Lim et al. 2015).

##### 2.4.4.2 Interoceptive Sensibility (task)

The second ANOVA analyzed the confidence ratings collected during the HCT. These scores define a state measure of interoceptive sensibility (ISen) construct because they are related to the current performance of the HCT. As independent variables, we again included *treatment* and *condition* as in the above ANOVA as well as *BMI* as a covariate.

As the exteroceptive condition was left‐skewed and not normally distributed, we repeated these two ANOVAs in a non‐parametric framework.

#### 2.4.5 Exploratory Analyses

##### 2.4.5.1 Interoceptive Sensibility (MAIA)

Interoceptive sensibility as the general belief in one's own interoceptive ability (ISen‐MAIA) was operationalized by means of the MAIA questionnaire (Mehling et al. 2012). This measure is unrelated to the performance of a specific interoceptive task and can thus be defined as a perceived trait. We specifically focused on three subscales investigating interoceptive aspects that relate to our study and tasks: Noticing (awareness of uncomfortable, comfortable, and neutral body sensations), Attention Regulation (ability to sustain and control attention to body sensations), and Body Listening (active listening to the body for insight). Group differences were calculated with two‐sample *t*‐tests.

##### 2.4.5.2 Interoceptive Awareness (task)

To investigate the metacognitive awareness of interoceptive abilities, we derived two measures. The first measure returns an individual index of interoceptive awareness (IAwa), calculated with a within‐participant Pearson correlation between the IAcc and ISen scores, as introduced by (Garfinkel et al. 2015). Since we acquired confidence ratings only every other trial (6 out of 12 total trials), we failed to balance this measurement between the interoceptive and exteroceptive conditions. Therefore, analytic constraints permitted only participants with at least three confidence ratings for the interoceptive condition to be included in the analysis. This led to a reduced sample of 56 participants (placebo group: *n* = 26; control group: *n* = 30) for this measure. Individual correlation coefficients were averaged to define group specific interoceptive awareness. Then, to determine if interoceptive awareness is present or absent (following the procedure of (Garfinkel et al. 2015)), a one‐sample *t*‐test was used to establish if the correlations between confidence and accuracy were significantly different from zero. This was performed on both the entire sample and on the two groups separately (resulting in a total of three one‐sample *t*‐tests). Finally, to assess if the placebo and control group differ in IAwa, we used a two‐sample *t*‐test.

Since this first measure of interoceptive awareness did not include all participants, we calculated a second measure which reflects the index of correspondence between subjective and objective accuracy at the group level and was calculated with a Pearson correlation between the individual mean of interoceptive accuracy scores and confidence ratings, separately for the placebo and control group. This method allowed us to include ratings from all participants, as well as the ones with fewer than three confidence ratings per condition. We then tested if the relationship between these dimensions differed in the groups by means of the test statistic *z*, comparing the two correlation coefficients with each other (see Figure 4).

##### 2.4.5.3 Bayesian Analyses

The surprising absence of any difference in the interoceptive dimensions between the two groups, going against our preregistered hypotheses, was followed up using a complementary Bayesian approach (e.g., (Wagenmakers et al. 2018)) that explored the relative evidence for either the null or the alternative hypothesis of no group difference. Four Bayesian two‐sample *t*‐tests were used to compare IAcc, ISen, ISen‐MAIA and IAwa between the two groups. A standard Cauchy (0.1) prior to 1 was used as the effect size. This refers to a 50% chance of observing an effect size that falls between −1 and 1 (e.g., Rouder et al. 2009). Bayesian *t*‐tests generate a Bayes Factor comparing the relative evidence between the alternative and null hypothesis (BF_10_, H1 vs. H0; Mac Giolla and Ly 2019). To interpret these values, it was proposed that a BF_10_ < 3 indicates weak evidence, a BF_10_ > 3 positive evidence, and a BF_10_ > 150 shows strong evidence for the alternative hypothesis (Jarosz and Wiley 2014). To investigate the evidence for the null compared to the alternative hypothesis (BF_01_, H0 vs. H1) the Bayes factor was calculated as BF_01_ = 1/BF_10_.

##### 2.4.5.4 Association Between Pain and Interoception

We tested the relationship between the analgesia score and the three interoceptive dimensions within both the total sample and the placebo and control groups separately by means of Pearson correlations.

##### 2.4.5.5 Sensitivity Analyses

We also repeated the above ANOVAs excluding people from the placebo group who had an analgesia score ≤ 0 (*n* = 4) as an exploratory sensitivity check. Moreover, as alexithymia is closely related to interoceptive ability (Murphy et al. 2018), the preregistered exclusion criterion of participants with a high TAS‐20 score may have risked artificially restricting variance in a construct central to our research question. We therefore re‐ran our main analyses for the three interoceptive dimensions without this criterion and by re‐including these five people in the dataset.

## 3 Results

### 3.1 Manipulation Checks

3.1.1

**FIGURE 2:**
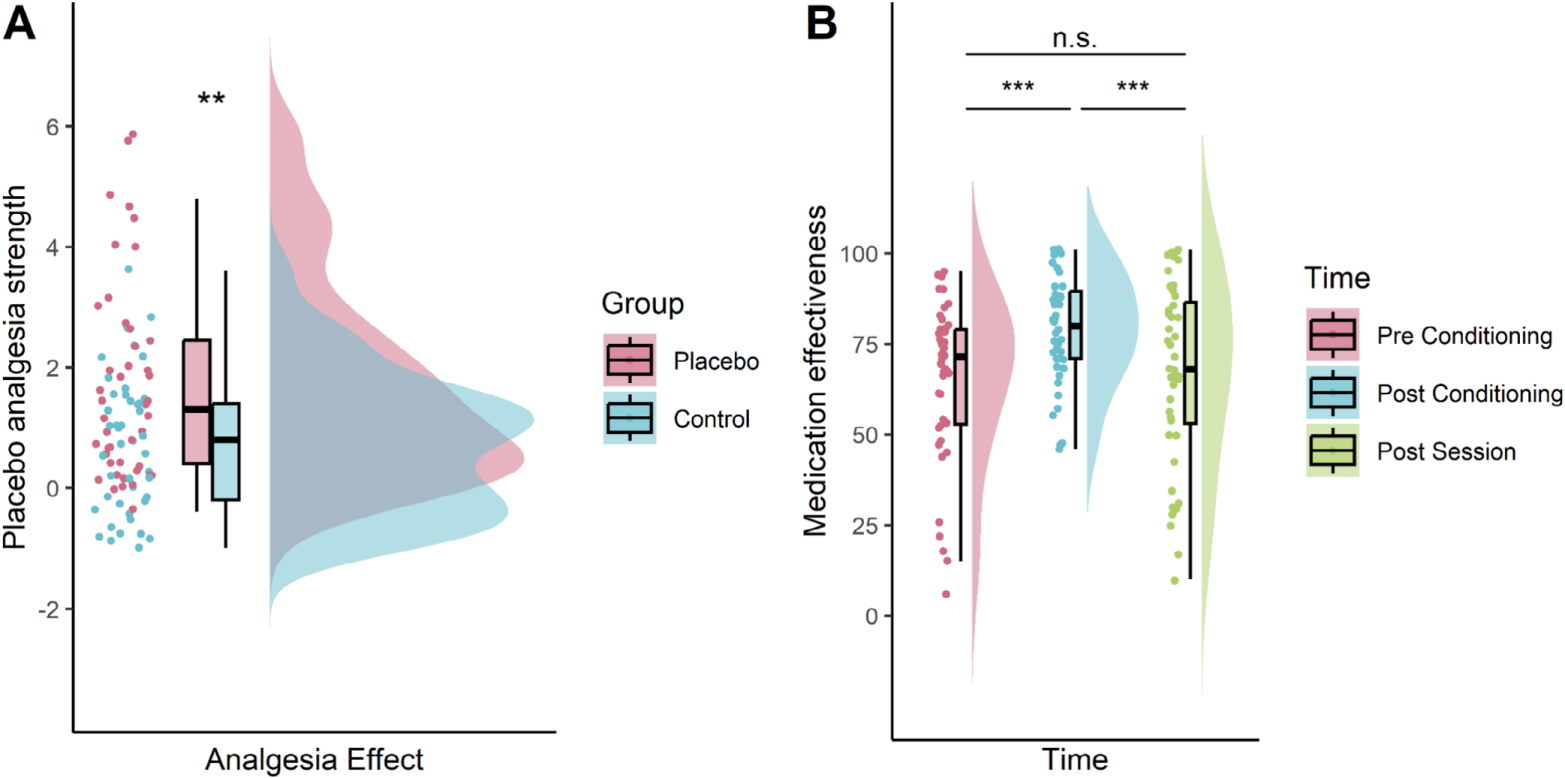
Manipulation checks of the placebo analgesia effect. (A) The placebo group showed a higher analgesia effect compared to a control group without such an induction. Participants who received placebo medication rated painful electrical stimuli as significantly less painful compared to participants who did not receive the medication. (B) Beliefs in the effectiveness of the administered alleged painkiller were rated by the placebo group at three time points during the experiment: after oral ingestion and verbal suggestions by the medical cover (pre‐conditioning), after the conditioning procedure (post‐conditioning) and during the debriefing at the end of the session (post‐session). This revealed a significant increase in beliefs after the conditioning. Although the beliefs significantly decreased until the end of the session, they did not drop significantly lower than initial belief levels (**p ≤ 0.01, ***p ≤ 0.001, n.s., not significant, *p* > 0.05). The boxes in the plots showed the interquartile range (IQR) of the data, where the upper and lower edges correspond to the first and third quartiles respectively. The horizontal line inside the boxes represents the median, while the whiskers outside the boxes indicate the range of non‐outlier data points (i.e., data not exceeding 1.5 times the IQR range). Raincloud plots are created based on the work of Allen et al. (2021).

First, we aimed to make sure that our placebo manipulation reliably reduced pain in the placebo group, compared to the control group. This check was not included in the preregistration, but we routinely employed it in previous studies (Hartmann et al. 2021, 2022). The placebo group reported a mean placebo analgesia score of 1.7 (SD = 1.64, max = 5.8, min = −0.4) while the control group had a mean of 0.7 (SD = 1.13, max = 3.6, min = −1), resulting in a strong group difference (𝑡(86) = 3.33, *p* = 0.001) with a medium to large effect size (*d* = 0.71). This effect was comparable to other placebo analgesia studies with the same setup (Rütgen, Seidel, Silani, et al. 2015, *d* = 0.79) and confirmed the validity of the placebo analgesia induction on subjective pain scores (see Figure 2A). When looking at the duration of the placebo effect, participants in the placebo group reported stronger beliefs of the effectiveness of the placebo pill in the post‐conditioning time point compared to the pre‐conditioning (𝑡(86) = 3.43, *p* < 0.001; both time points before the HCT). At the end of the session (post‐session time point, after the HCT) the beliefs of the medication effectiveness were significantly reduced compared to the post‐conditioning phase (*t*(86) = 2.70, *p* = 0.008), but still rather high overall, and not lower than the initial pre‐conditioning belief (𝑡(86) = 0.37, *p* = 0.715) (see Figure 2B).

### 3.2 Control Variables

#### 3.2.1 BMI

The total sample reported a BMI average of 22.8, falling into the range of what is considered healthy body weight (Peterson et al. 2016; see Table 1). No significant difference was found between the placebo and control group (*t*(86) = −0.52, *p* = 0.603; placebo: *M* = 22.62, SD = 2.55; control: *M* = 22.96, SD = 3.48).

#### 3.2.2 Clinical Questionnaires

We administered several questionnaires assessing personality traits that could be related to pain processing or interoception, to make sure our two groups were comparable. Between the placebo and control group, no significant differences were found in the scores of all questionnaires administered (see Table 2 for the complete list) (all *p's* > 0.126, before and after applying Bonferroni correction for multiple comparisons). In the total sample and in the placebo group, there was no significant relationship between the interoceptive dimensions and a measure of depression (BDI), alexithymia (TAS) and anxiety (STAI‐T) (total sample: *r* < ±0.17, all *p's* > 0.104, placebo group: *r* < ±0.27, all *p's* > 0.073). In the control group, a negative trend was evident between alexithymia and interoceptive sensibility (i.e., confidence ratings) (*r* = −0.29, *p* = 0.054).

#### 3.2.3 Post‐Session Questionnaires

No group difference was found in the evaluation of the difficulty of the interoceptive and exteroceptive conditions of the HCT (*t*(86)'s < 0.96, *p's* > 0.337). However, all participants rated the interoceptive condition significantly more difficult than the exteroceptive one (*t*(174) = −29.78, *p* < 0.001), which indicates that the difficulty level between the two conditions was not correctly balanced. No group differences were found when asking if the counted heartbeats were felt in a specific location or generally in the body (*t*(86)'s < 1.34, *p's* > 0.184). Regarding the strategies used to count heartbeats, both groups reported a similar level of guessing and estimation strategies whenever they didn't feel any heartbeat (*t*(86)'s < 1.45, *p's* > 0.151) (guessing strategies: placebo: *M* = 1.23, SD = 0.47; control: *M* = 1.45, SD = 0.93; estimation strategies: placebo: *M* = 1.57, SD = 0.95; control: *M* = 1.64, SD = 1.01). The little use of these strategies implies good compliance with the amended task instructions.

### 3.3 Preregistered Results

#### 3.3.1 Interoceptive Accuracy

**FIGURE 3:**
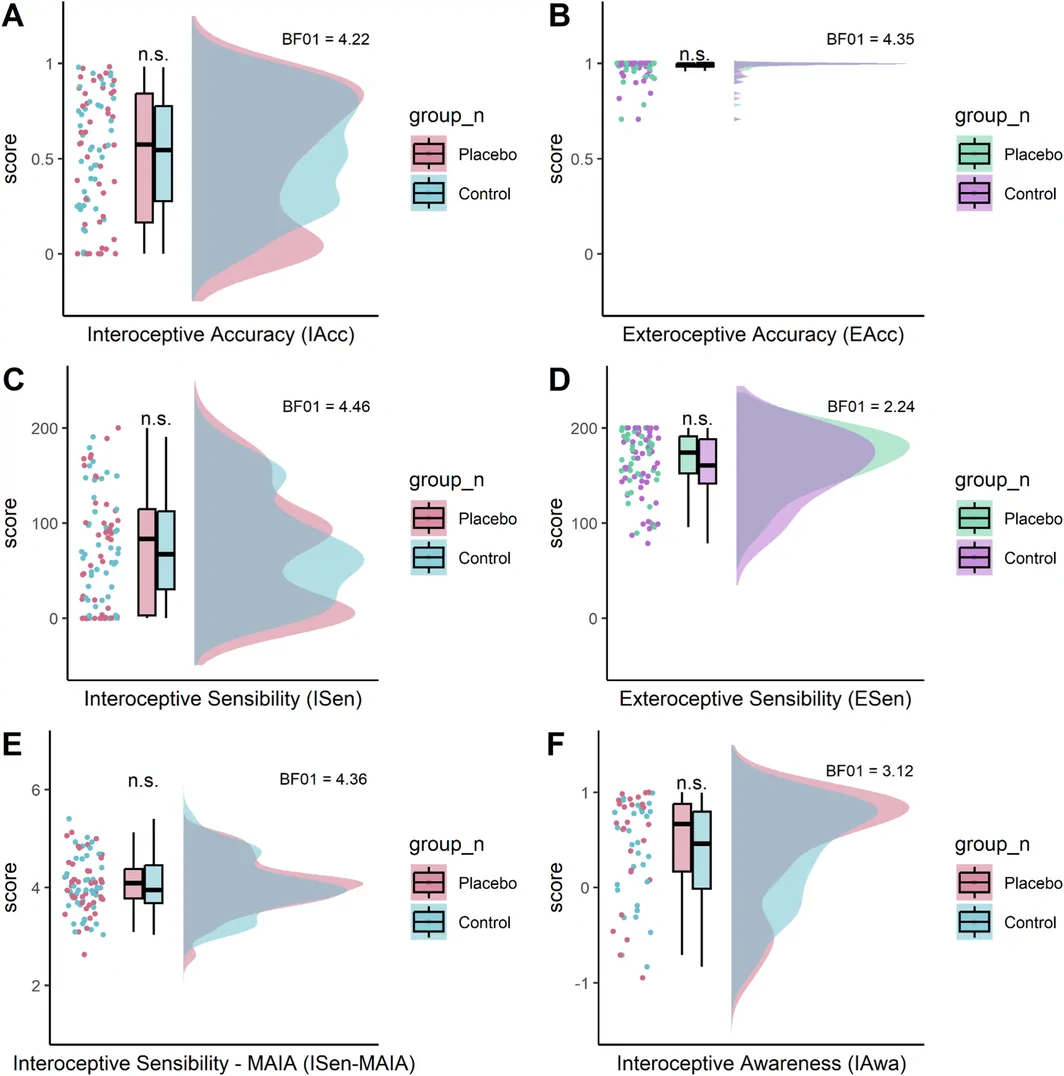
No effects of placebo analgesia on the four dimensions of interoception. (A) Interoceptive accuracy (objective performance in the heartbeat counting task). (B) Exteroceptive accuracy. (C) Interoceptive sensibility (the subjective perception of one's performance in the heartbeat counting task). (D) Exteroceptive sensibility. (E) Interoceptive sensibility (general subjective perception of one's interoceptive abilities in a questionnaire). (F) Interoceptive awareness (the correlation between objective performance and its subjective perception). Corresponding Bayes Factors (BF) are indicated in the top right corner of each plot. All BFs were higher than 3 in the interoceptive dimensions indicating positive evidence for the null hypothesis (i.e., no group difference in the interoceptive dimensions). For the exteroceptive dimensions, the exteroceptive accuracy also showed a BF higher than 3, while for exteroceptive sensibility, the value was 2.24 indicating anecdotal evidence for the null hypothesis. The boxes in the plots showed the interquartile range (IQR) of the data, where the upper and lower edges correspond to the first and third quartiles respectively. The horizontal line inside the boxes represents the median, while the whiskers outside the boxes indicate the range of non‐outliers data points (i.e., data not exceeding 1.5 times the IQR range). n.s., not significant (*p* > 0.05), BF_01_ = evidence for the null relative to the alternative hypothesis.

The total sample reported for the interoceptive condition and the exteroceptive condition a mean accuracy score of 0.52 (SD = 0.32, max = 0.98, min = 0) and 0.97 (SD = 0.06, max = 1, min = 0.7) respectively. No group difference in the HCT performance was found in the main effect of group (*F*(1, 86) = 3.21, *p* = 0.116, 𝜂̂^2^_𝐺_ = 0.198) (see Figure 3A), neither in the group × condition interaction (*F*(1, 86) = 3.12, *p* = 0.121, 𝜂̂^2^_𝐺_ = 0.170). Instead, a strong difference between conditions was found (main effect of condition: *F*(1, 86) = 251.93, *p* < 0.001, 𝜂̂^2^_𝐺_ = 0.943), with an overall better performance for the exteroceptive condition compared to the interoceptive condition. We observed a ceiling effect (36.36% of participants had a perfect score, i.e., an accuracy of 100%) for the exteroceptive condition which indicates a disproportion in the difficulty level between the two conditions (similar as in other studies, e.g., Sojka et al. 2024).

#### 3.3.2 Interoceptive Sensibility

There was no group difference in the confidence ratings (*F*(1, 86) = 0.05, *p* = 0.827, 𝜂̂^2^_𝐺_ = 0.006) (see Figure 3B) and the group × condition interaction was found not significant (*F*(1, 86) = 2.91, *p* = 0.132, 𝜂̂^2^_𝐺_ = 0.065). Like above, only the difference between conditions was significant (*F*(1, 86) = 415.99, *p* < 0.001, 𝜂̂^2^_𝐺_ = 0.908), meaning that participants were more confident about their exteroceptive performance compared to the interoceptive performance in the HCT. In the non‐parametric ANOVA, the results stayed largely the same, except for an additional interaction between condition × BMI (*F*(1, 86) = 3.90, *p* = 0.031, 𝜂̂^2^_𝐺_ = 0.826), showing that BMI was negatively associated with confidence ratings in the interoceptive condition, but not in the exteroceptive one.

The results of both analyses did not change in the non‐parametric ANOVAs.

### 3.4 Post Hoc Results

#### 3.4.1 Interoceptive Sensibility—Trait

Interoceptive sensibility as a trait was operationalized by means of the MAIA questionnaire. Placebo and control groups did not differ in the total score (86 = −0.25, 𝑝 = 0.79) (see Figure 3C) as well as in the subscales of interest (Noticing, Attention Regulation, and Body Listening) (𝑡(86) < ±1.39, *p's* > 0.17).

#### 3.4.2 Interoceptive Awareness

We found a significant positive correlation between confidence ratings and interoceptive accuracy in both the total sample (*r* = 0.38, *p* < 0.001) and in the placebo (*r* = 0.35, *p* = 0.019) and control group (*r* = 0.42, *p* = 0.004).

**FIGURE 4:**
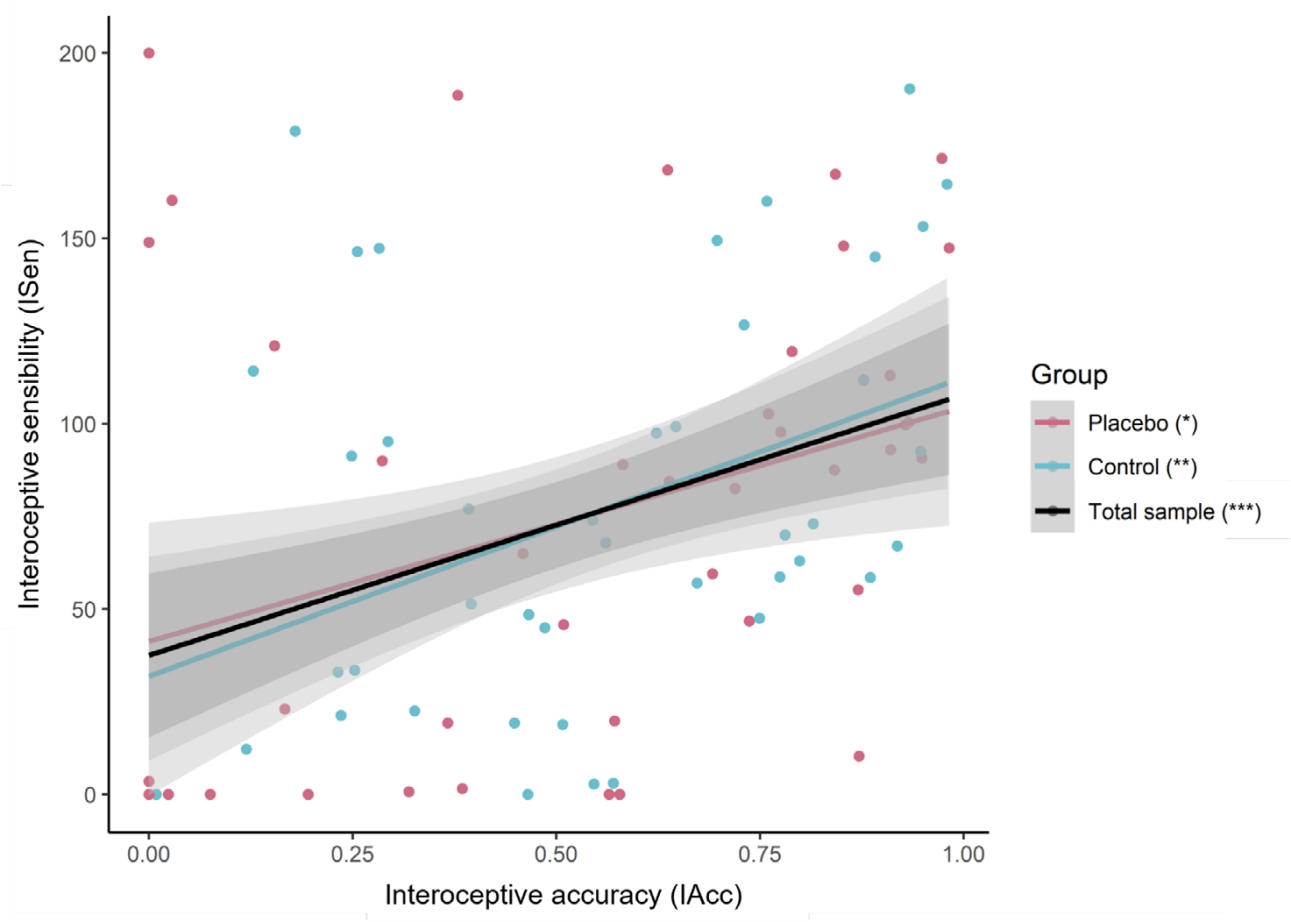
*C*orrespondence between subjective and objective interoceptive abilities at the group level in the placebo and control group as well as for the whole sample. The total sample showed a significant relationship between interoceptive accuracy and interoceptive sensibility (*r* = 0.38, *p* < 0.001) as well as the placebo group (*r* = 0.35, *p* = 0.019) and the control group (*r* = 0.42, *p* = 0.004). However, no group difference was found when directly comparing the two correlation coefficients (Fisher's *z* = −0.40, *p* = 0.689). The black line represents the average line between the two groups (*p ≤ 0.05, **p ≤ 0.01, ***p ≤ 0.001).

However, the correlation coefficients did not differ between the two groups (Fisher's *z* = −0.40, *p* = 0.689), indicating that the correspondence between subjective and objective interoceptive abilities in the placebo and control group is not statistically different (Figure 4). Individual interoceptive awareness determined from confidence‐accuracy correlation (Pearson's *r*) significantly differed from zero at the total sample level (56 participants, (55) = 5.30, *p* < 0.001) and in the placebo (26 participants, *t*(25) = 3.90, *p* < 0.001) and control group (30 participants, *t*(29) = 3.56, *p* = 0.001). When comparing interoceptive awareness between the two groups, no significant difference was found (*t*(54) = 0.52, *p* = 0.603) (see Figure 3D).

#### 3.4.3 Bayesian Follow‐Up Analyses

The absence of group difference in all three interoceptive dimensions (i.e., interoceptive accuracy, interoceptive sensibility, and interoceptive awareness) was further explored with Bayesian two‐sample *t*‐tests for each dimension separately. All results showed moderate evidence against the alternative hypothesis, with the null hypothesis being three to four times more likely than the alternative hypothesis in our sample (IAcc: BF_01_ = 4.22; ISen: BF_01_ = 4.46; ISen‐MAIA: BF_01_ = 4.36; Iawa: BF_01_ = 3.12). Furthermore, moderate evidence against the alternative hypothesis was found also for the exteroceptive condition in the HCT (BF_01_ = 4.35), while the related confidence ratings showed anecdotal evidence (BF_01_ = 2.24). However, the findings for the exteroceptive condition have to be interpreted with caution in light of the observed ceiling effects.

#### 3.4.4 Association Between Pain and Interoception

No significant correlations were found between the placebo analgesia effect and the interoceptive dimensions in both the total sample and in the placebo and control group separately (*r* < ±0.17, all *p's* > 0.265).

#### 3.4.5 Sensitivity Analyses

The results of the two above ANOVAs did not change when excluding the four people who showed an analgesia score ≤ 0 in the task. The findings also did not change when re‐including five participants with TAS‐20 scores above the cut‐off (all *p*'s > 0.116).

## 4 Discussion

We investigated whether placebo analgesia, the reduction of pain by means of a placebo pill presented as a “strong painkiller” and a related conditioning procedure, would generalize to cardiac perception. Using a combination of frequentist and Bayesian statistical approaches, we observed consistent moderate evidence of absence for such a transfer in all three interoceptive dimensions.

This evidence for an absence of effects was observed even though participants in the placebo group felt significantly less pain than those in the control group after the manipulation and the belief in this pain reduction lasted until the end of the experiment. The two groups did not differ in their interoceptive accuracy, that is, their objective performance of counting their heartbeats. They also did not differ in their interoceptive sensibility, that is, how confident they felt in correctly counting their heartbeats during a cardiac interoceptive task and how they rated their general interoceptive ability in a self‐report trait questionnaire, respectively. Similarly, the two groups did not show differences regarding their interoceptive awareness, that is, how well people's subjective perception and objective performance matched in the counting task. At the group level, we did find that both the placebo and control group showed a significant positive correlation between confidence reports and task performance. This correlation indicated that people who were more accurate in counting their heartbeats were also more confident in their performance, or vice versa. However, the correlation coefficients of the two groups were not different when statistically compared. These null findings in the three interoceptive dimensions were corroborated by moderate Bayesian evidence for the null hypothesis (which was 3–4× more likely than the alternative hypothesis), and further evidenced by the absence of a significant correlation between the placebo analgesia effect score and the measure of any interoceptive dimension.

Our findings are in line with the study of Werner et al. (2009) who reported no difference between participants with high and low interoceptive sensitivity regarding pain sensitivity, pain tolerance, and subjective pain experience of tonic heat stimuli. Also, they found no significant correlation between pain sensitivity and cardiac interoceptive sensitivity. According to some accounts, pain applied on the skin might also be considered an exteroceptive sensation (Ceunen et al. 2016). These findings possibly highlight the limits and extent to which our own pain processing may affect heartbeat perception, proposing that distinct mechanisms are at play. Perceiving one's heartbeat might rely, in presumably large parts, on different underlying mechanisms than pain perception, even though some neural processing might rely on similar brain regions such as the insula (Legrain et al. 2011). Here, we tested the influence of reduced pain perception via a placebo on interoceptive abilities derived from self‐reported measures in a conscious heartbeat tracking task. Future studies should validate these findings with objective data, for instance by using fMRI to compare neural activity in key interoceptive regions during interoceptive tasks between the two groups.

Perceiving one's heartbeats is only one facet of interoception, and we cannot generalize our findings to other, more internal or unconscious forms of interoception (e.g., visceral or gut interoception, Smith et al. 2021; or unconscious interoception (Azevedo et al. 2017)). Moreover, it could also be that people with better interoceptive abilities feel pain more intensely and are thus more sensitive or accurate when perceiving pain. This notion, however, will have to be tested in future work, for example, by strategically collecting and comparing data from individuals “better” or “worse” at interoception.

In connection with this, research has shown that placebo analgesia also reduces the first‐hand experience of unpleasant, but not pleasant, touch (Rütgen et al. 2021). This suggests a valence‐specific categorization and modulation of incoming sensations into positive and negative. Our results extend this knowledge to the more neutral domain of heartbeat perception by showing that top‐down pain reduction does not generalize to such neutral body sensations. However, this does not exclude the possibility that placebo analgesia may generalize to states of higher arousal, such as the perception of negative affect.

These findings seem intuitive from an evolutionary standpoint: while some generalizations of bodily perception may be helpful, they may be overly cautious in other cases: distinguishing heartbeat perception from the perception of pain is useful, as especially the latter serves as a warning and warrants concrete action. Moreover, while heartbeats are a signal that is automatically regulated by the body, acute pain often demands conscious attention, at least in otherwise healthy individuals. The picture might look different in chronic pain patients, where pain has lost its warning function and has been shown to be related to interoceptive conditions and sensations (Di Lernia et al. 2016 for a review; Borg et al. 2018 for fibromyalgia; Grabli et al. 2021 for back pain; Weiss et al. 2014 for somatoform disorder). Future work should also focus on further teasing apart the connection between increased versus decreased pain and interoception in health versus disease.

The present study had some strengths and limitations worth mentioning. We successfully induced a medium to large placebo analgesia effect in the placebo group, as they rated electrical pain received on their hand as significantly less painful compared to the control group, who did not receive any treatment. The placebo group also had a high belief in the “medication”, which significantly increased through the conditioning procedure and remained present until the end of the session. This validates our manipulation and strengthens the evidence of absence of a transfer of placebo analgesia to heartbeat perception. The interoception task was done around 45 min after the placebo analgesia induction, which could have decreased transfer effects and limits causal conclusions. On the other hand, participants were told that the effects of the “painkiller” would stay stable until the end of the experiment, and we observed transfer effects to the task measuring prosocial behavior completed right before the HCT (Hartmann et al. 2022). Nevertheless, future work should replicate our findings in a design where interoceptive abilities are measured during or directly after the placebo manipulation and interoception is the main focus of the study. Relatedly, it would be interesting to next test the causal effects of placebo analgesia on cardiac perception during acute pain administration.

One potential concern regarding the choice and dosage of the substance used in the placebo pills (Mannitol, 40 g per pill) is worth mentioning. Although this substance was selected in agreement with the university's pharmacy and participants were accurately screened as healthy without medical conditions, Mannitol is reported to potentially induce gastrointestinal side effects like bloating, cramping, and diarrhea at high doses (Tuck et al. 2014; Yao et al. 2014). These side effects might introduce physiological noise that could affect performance during the interoception task. While only four participants reported experiencing bloating, we cannot completely rule out an impact on the results. Therefore, we suggest that further studies consider using a substance without such side effects as a placebo, to corroborate our findings. Additionally, future interoception‐related work might incorporate validated tools for gastrointestinal disorders (e.g., the Rome IV criteria questionnaire or Irritable Bowel Syndrome Symptom Severity Score) as part of the screening process to be able to exclude individuals at higher risk of gastrointestinal side effects.

The groups were comparable regarding their BMI and personality traits such as depression, alexithymia, and anxiety, and the inclusion of these variables as covariates did not change the overall findings. This homogeneity of the two groups regarding sociodemographics and trait measures allows for increased confidence in the observed results, especially since the Bayesian analyses showed moderate Bayes Factors above three. However, as these and other analyses in the study were done post hoc rather than preregistered, replication in independent studies is warranted.

Moreover, participants were asked to abstain from caffeine and nicotine 1 h before the experiment. However, both nicotine and caffeine have longer half‐lives of two (Hukkanen et al. 2005) and 4 h (Alsabri et al. 2018), respectively. This means that residual effects of such substances could still have influenced heart rate and autonomic nervous system activity, and thus interoceptive performance. However, since the interoception task was conducted last in the whole study, about 2.5 h after participant arrival, this actually adds up to a minimum of ~3.5 h abstinence, since participants only drank water during the experiment. We are therefore confident that these substances had a negligible effect on our task. Future studies should nevertheless add a longer abstinence period and/or measure individual substance use before the experiment.

We screened and excluded participants with chronic, CNS‐related, neurological, and/or psychiatric conditions, but did not explicitly include cardiovascular diseases as an exclusion criterion. Although the impact of cardiovascular diseases is low in our healthy, young student sample, they may influence people's performance in the HCT and should be directly measured and controlled for in future work.

We further observed that participants complied with task instructions and completed the task as intended. Counting strategies such as guessing or estimating were similar in both groups. However, the criticism which has been voiced regarding the HCT cannot be overlooked (but see Ainley et al. 2020; Christensen et al. 2021). We adapted the task instructions to mitigate some of its criticism, but future studies should turn to more valid, updated measures of interoception to replicate our (absence of) findings (see e.g., Desmedt et al. 2025 and Murphy et al. 2019 for reviews). While the nature of the task employed in this study required a focus on heartbeats extracted from the heartrate, other measures such a heart rate variability are gaining in popularity as an indicator of autonomic regulation (see e.g., Larsson et al. 2021; Lischke et al. 2021). Here, however, we abstained from its analysis due to our preregistered analysis plan.

Interestingly, Körmendi et al. (2021) reported that the HCT is sensitive to performance‐related expectations. As we did not explicitly measure such expectations, it would be interesting to conduct a future study where people are asked to report their expected performance or primed with different expectations regarding the task, for example, that the “painkiller” may also have effects on heartbeat perception.

Moreover, the interoceptive and exteroceptive conditions were unbalanced in terms of subjectively perceived difficulty. Although we tried to match the two conditions as best as possible using an elaborate sound calibration procedure to ensure the tone volume was just perceivable (as described by Pollatos et al. 2007), ~36% of participants had the highest possible accuracy in the exteroceptive condition. We also observed higher confidence when counting tones versus heartbeats over all participants, which matched the accuracy results. This imbalance may have contributed to participants rating the interoceptive condition as more difficult and to the exteroceptive condition showing a ceiling effect. These observations weaken the validity of using tone counting as an exteroceptive task and control. They could further indicate that differences between conditions may be related to a different level of allocation of attentional resources or task challenge rather than interoceptive ability, although this allocation seemed to be similar for our two groups. It is important to note that there has been considerable debate and discrepancy in the literature regarding the selection of an appropriate control measure, which is why different studies have employed different measures (Murphy 2024). Moreover, an imbalance in difficulty between the interoceptive and exteroceptive conditions has been observed in other studies using similar interoceptive tasks (Salamone et al. 2020; Yoris et al. 2017). Future work should therefore focus on using more ambiguous signals to better match interoceptive and exteroceptive conditions.

Relatedly, literature indicates that auditory stimuli can modulate interoceptive accuracy. For example, Canales‐Johnson et al. (2015) found that auditory feedback of one's heartbeat improved later accuracy of synchronous tapping to one's heartbeat. Haruki and Ogawa (2024) reported that tones played during heartbeat counting diminished interoceptive accuracy, confidence, and intensity. With these findings in mind, we cannot fully exclude the possibility that the exteroceptive condition may have influenced performance in the interoceptive condition, as they were pseudorandomly alternated in the task. These potential attentional effects have to be considered when interpreting the results, and future studies may choose alternative control conditions that are more independent from the interoceptive performance.

Another task‐related limitation regards the different proportion of confidence rating measures across the participants. Since they were assessed in every other trial no matter the condition, we failed to balance this measurement between the interoceptive and exteroceptive conditions, resulting in the exclusion of some subjects from the analyses on interoceptive awareness. Moreover, placebo analgesia has been suggested to act mainly via higher‐level regulatory mechanisms, as opposed to pain (Zunhammer et al. 2021), which could be another reason why we did not observe any effects in this study. Due to our preregistered nonresponder criteria, we did not exclude participants based on their placebo effects in the pain task. This variability could have muddled transfer effects we might have observed in strong placebo responders. However, excluding people who did not have a placebo effect in the pain task in a post hoc analysis did not alter the results.

In conclusion, we show moderate and consistent evidence of the absence of a transfer of pain reduction by means of placebo analgesia to the objective and subjective perception of heartbeats. Studies such as the present one are important to better understand the perception of different bodily systems and their connection in humans.

## Author Contributions

**Alessio Proposito:** formal analysis, validation, visualization, writing – original draft, writing – review and editing, data curation. **Claus Lamm:** conceptualization, supervision, funding acquisition, writing – review and editing, resources, methodology. **Federica Riva:** conceptualization, supervision, writing – review and editing, methodology. **Helena Hartmann:** conceptualization, methodology, software, data curation, investigation, formal analysis, funding acquisition, visualization, supervision, project administration, writing – original draft, writing – review and editing.

## Funding

The work was supported by the uni:docs scholarship (awarded to HH) of the University of Vienna, the Austrian Science Fund (FWF W1262‐B29), and the Vienna Science and Technology Fund (WWTF VRG13‐007). HH was additionally supported by the Deutsche Forschungsgemeinschaft (DFG, German Research Foundation—Project‐ID 422744262 ‐ TRR 289). None of the funders had any role in study design, data collection and analysis, interpretation, writing or decision to publish. The authors declare that they have no financial interests or potential conflicts of interest.

## Conflicts of Interest

The authors declare no conflicts of interest.

## Data Availability

The data that support the findings of this study are openly available in the Open Science Framework at https://osf.io/fz73k/.
